# Authorship Policies at U.S. Doctoral Universities: A Review and Recommendations for Future Policies

**DOI:** 10.1007/s11948-020-00273-7

**Published:** 2020-11-19

**Authors:** Lisa M. Rasmussen, Courtney E. Williams, Mary M. Hausfeld, George C. Banks, Bailey C. Davis

**Affiliations:** 1 Department of Philosophy and Graduate School Faculty Fellow, University of North Carolina Charlotte, 9201 University City Blvd., Charlotte, NC 28223 USA; 2 Department of Management, University of Toledo, 2801 Bancroft St., Toledo, OH 43606 USA; 3 Department of Management, Belk College of Business, University of North Carolina Charlotte, 9201 University City Blvd., Charlotte, NC 28223 USA; 4 Department of Ethics and Compliance, Memorial Health University Medical Center, 4700 Waters Ave, Savannah, GA 31404 USA

**Keywords:** Authorship, Institutional authorship policy, Authorship dispute resolution

## Abstract

Intellectual contribution in the form of authorship is a fundamental component of the academic career. While research has addressed questionable and harmful authorship practices, there has largely been no discussion of how U.S. academic institutions interpret and potentially mitigate such practices through the use of institution-level authorship policies. To gain a better understanding of the role of U.S. academic institutions in authorship practices, we conducted a systematic review of publicly available authorship policies for U.S. doctoral institutions (using the 266 2018 Carnegie-classified R1 and R2 Universities), focusing on components such as specification of authorship criteria, recommendations for discussing authorship, dispute resolution processes, and guidance for faculty-student collaborations. We found that only 24% of the 266 Carnegie R1 and R2 Universities had publicly available authorship policies. Within these policies, the majority (93%) specified criteria for authorship, but provided less guidance about actual processes for applying such criteria (62%), handling authorship disputes (62%), and managing faculty-student author teams (49%). Further, we found that any discussion of dispute resolution practices typically lacked specificity. Recommendations grounded in these findings are offered for institutions to leverage their ability to guide the authorship process by adopting an authorship policy that acknowledges disciplinary diversity while still offering substantive guidance.

## Introduction

The importance of intellectual credit in the form of authorship in published research has been acknowledged for decades. Authorship is frequently called “the coin of the realm” in academia (Rennie and Flanigan 1994; Wilcox 1998; Macrina 2011), and authorship standards in various disciplines and journals multiply with each passing year (Rennie Yank and Emanuel 1997; Tscharntke et al. 2007; Silva et al. 2016a, b; Clement 2014; Tarkang et al. 2017). Despite sustained attention, ambiguity around authorship decisions, conflict, and even abuse, continues (Smith et al. 2019a; Marušić et al. 2011; Birnholtz 2006; Gómez-Ferri et al. 2019).

As the front line of the publication process, journal editorial offices have recognized their role in addressing guidance on authorship by offering standards of authorship, especially via international editorial organizations that have disseminated authorship standards for years (ICMJE n.d.^1^;^2^; COPE 2016; APA 2020; Editors 2009). Much has been written about *ghost* authorship (i.e., excluding someone who made an intellectual contribution), *gift* authorship (i.e., including someone who did not make an intellectual contribution), and *guest* authorship (i.e., adding a senior author who did not make an intellectual contribution) in particular, with consensus in condemning these practices (Bosch et al. 2012; Lacasse and Leo 2010; Sismondo and Doucet 2010). Similarly, many articles recommend methods for discussing authorship among a team (Frassl et al. 2018; Jones and Cairns n.d.; McNutt et al. 2018; Primack et al. 2014; Roig 2007; Sandler and Russell 2005; Smith and Master 2017; Street et al. 2010), and offer frameworks for acknowledging authorship based on universally recognized standards such as ICMJE (n.d.^1^;^2^), COPE (2016), or APA (2020).

While journals disseminate standards of authorship, methods for facilitating transparent authorship conversations and resolving conflicts have received much less attention (Faulkes 2018). Journal editors do not seem inclined to resolve such problems; at least one international editorial organization has explicitly indicated: "it is not the role of journal editors to determine who qualifies or does not qualify for authorship or to arbitrate authorship conflicts. If agreement cannot be reached about who qualifies for authorship, the institutions where the work was performed, not the journal editor, should be asked to investigate." (ICMJE n.d.^2^, II.A.2.4). Up to this point, however, there has been little to no discussion or data regarding how U.S. academic institutions interpret or execute this responsibility. This creates a potential problem: if journals defer to institutions to resolve authorship conflict, and we have no sense of how institutions take up that responsibility, then we have no sense of how authorship disputes are prevented or resolved.

In particular, it is unclear to what extent common practices exist among institutions for addressing authorship disputes. If research-intensive institutions, from which most research grants and publications stem, are not providing explicit guidance and leadership regarding preventing and resolving authorship disputes, then the issue is falling to individual teams and researchers to address. It is challenging to achieve fairness and transparency in authorship determinations when the most problematic cases are resolved in private. Further, under these circumstances, collaborators with less power will likely suffer the most from *sub-rosa* authorship conflict resolutions (Faulkes 2018). Data on institutional policies is critical for improving the recognized problem of authorship conflict and abuse.

In the current work, we set out to determine:

1. The prevalence of authorship policies among U.S. Doctoral institutions
2. The characteristics of such policies
3. The prevalence among existing policies of processes for handling authorship disputes
4. The characteristics of dispute resolution processes
5. Common practices and takeaways
6. Areas needing additional discussion and analysis
7. Recommendations for future policies

## Literature Review

If authorship is the coin of the academic realm, it has two equally important sides: credit and responsibility (Rennie and Flanagin 1994). Inclusion of one’s name as an author usually requires, at minimum, that one has made a substantial intellectual contribution to the research. In our review, we define authorship as a signal of credit and responsibility for the production of work (Chang 2019; Smith and Master 2017). Authorship is related to but distinct from the notion of contributorship, which is meant to acknowledge contributions to a work that do not rise to the level of authorship. Thus, authorship declarations on manuscripts describe “general tasks, including, study design, data collection, analysis and writing” (Smith and Master 2017: 244). The determination of authorship and contributorship depends on the academic discipline (Frandsen and Nicolaisen 2010). We focus specifically on authorship in our review.

The value of authorship has inflated with the increasing importance of publications and the competition for academic positions (Papatheodorou et al. 2008). A predictable outcome of these circumstances is disputes over authorship credit, including both being listed as an author and where one’s name appears on that list. Research attention has often focused on what constitutes authorship, how to determine author order, and how to make it transparent in journal publications (Brand et al. 2015; McNutt et al. 2018). International organizations of publishers and editors, such as the International Council of Medical Journal Editors (ICMJE), the Committee on Publication Ethics (COPE), and the World Association of Medical Editors (WAME), have disseminated consensus standards for authorship attribution and model policies for journals to facilitate transparency and clarity (ICMJE n.d.^1^;^2^; COPE 2016; WAME 2007). Authorship is also a commonly recognized content area in courses on the responsible conduct of research.

Despite these measures, problematic authorship practices persist. For example, a recent Wellcome Trust poll investigated perceptions of research culture from 4065 participants, with the majority of participants conducting research in academia/universities (84%). They found that “40% of all respondents said that they had experienced issues with others taking credit for their work” (Wellcome Trust 2020, 33). Clearly, authorship disputes are predictable and relatively common, even amidst the proliferation of well-publicized authorship standards. Further, the increasing occurrence of interdisciplinary, international authorship groups without common authorship practices may increase the number of disputes in the future (Smith and Masterson 2017).

Two additional practices in academic publication highlight the policy gap into which authorship disputes fall. First, following federal policy, institutions commonly exclude authorship disputes from counting as research misconduct but without offering alternative policies to cover those disputes. Second, publishers and journals recommend that institutions, rather than editorial offices, arbitrate such disputes (e.g., ICMJE n.d.^2^; II.A.2.4 as referenced above). The database of authorship cases submitted to the Committee on Publication Ethics (COPE) for guidance demonstrates that COPE similarly recommends to member journals that they refer authorship disputes back to research institutions for resolution (see, e.g., cases 16-15, 06-12, 98-02, 15-06). Thus, two of the most likely places to which one might turn for resolution of authorship disputes explicitly disavow any role in such resolution. This leaves the responsibility for authorship dispute resolution squarely with the institution, but not subject to research misconduct policies.

There are, in addition, two principled reasons for institutions to address authorship issues. First, authorship practices—particularly disagreements—likely contribute to the culture of research integrity at an institution. For example, recent work has shown a correlation between authorship conflicts and other research misbehavior (Smith et al. 2019a, (b). TheNational Academies of Science, Engineering, and Medicine (NASEM) Report *Fostering Integrity in Research* counted authorship as an important component of research integrity, and observed that “several of the most difficult challenges to research integrity involve authorship abuses, particularly authorship credit misallocations/misappropriations” (2017, 138). The report recommends that "research institutions can make an important contribution to stronger authorship standards. The widespread development and dissemination of [authorship] standards will make a significant contribution to research integrity" (NASEM 2017, 156). As both the site of research and the institution in which research integrity practices and culture are housed, universities have an important role to play regarding authorship.

Second, the lack of explicit and regular conversation about authorship decision making within institutions may disadvantage vulnerable contributors, such as graduate students or junior faculty, disproportionately (Feldon et al. 2017; Fine and Kurdek 1993; Welfare and Sackett 2010; Kwok 2005). Female students and faculty may also receive less credit for their contributions (Macaluso et al. 2016; Symonds et al. 2006). Moreover, these unethical practices make it likely that students learn implicit lessons about assigning authorship via a “hidden curriculum” (Fryer-Edwards 2002; Hafferty 1998). Theories about culture may help to explain this phenomenon: “organizational cultures often implicitly encourage while simultaneously condemning misconduct, motivating members to achieve particular ends without providing guidance on how these should be achieved” (Hall and Martin 2019, 3). To the extent that institutions wish to change their research culture, addressing confusion or disputes about authorship is a strong candidate for targeted interventions.

Thus, multiple arguments suggest that institutions should take the lead on cultivating appropriate authorship practices, particularly dispute resolution mechanisms. The Australian Government has instituted such a practice: In 2007, the Australian Code for the Responsible Conduct of Research was published, mandating an institutional responsibility for establishing policies regarding authorship standards and dispute procedures: “Institutions must have a policy on the criteria for authorship consistent with this Code, seeking to minimise disputes about authorship and helping to resolve them if they arise” (NHMRC, ARC and UA 2007, Sect. 5.1). (A 2018 policy revision (NHMRC, ARC and UA 2018) was accompanied by a separate guide on Authorship (NHMRC, ARC and UA 2019).) Two years after the initial policy was published, Morris (2010) evaluated institutional compliance with the Code, finding that the majority of Australian institutions (29 out of 39) were substantially or entirely compliant with the Code. No comparable data exist regarding institutional policies concerning authorship disputes in the United States; we set out to address this gap.

## Methods

### Data Collection Procedures

Our data sources for this review were websites of U.S. Doctoral institutions identified in the Carnegie Classification of Institutions of Higher Education (2018) as R1 (https://tinyurl.com/y7t6d6gj) or R2 institutions (https://tinyurl.com/yde9g7w5). This resulted in a total of 131 R1 institutions and 135 R2 institutions, with a combined count of 266 institutional websites as potential data sources. We used the following systematic search procedures to collect data from each institution’s website. First, we accessed each institution’s website via a standard Google search. Second, we used the institution’s homepage “search” feature to search for authorship-related terms, including: "authorship policy", "authorship agreement", “authorship dispute”, “authorship guidelines”, "author policy", "author agreement", "author dispute", “guidelines on authorship”, and “guidelines for authorship.” Third, we reviewed the results of the search for anything related to authorship guidance.

Any links or documents that conveyed authorship guidance from an institutional level were retained for coding. We conceptualized the institutional level as the college-and school-level or above, with a requirement that the college or school must have departments within it. In other words, if a college or school did not have departments within it, then it was not considered higher-level authorship guidance. As a final check for thoroughness, we completed the search process delineated above for a second time to account for any potential missing data. This secondary search took place in fall of 2019 and, as such, our data represents institution-level authorship guidance presented to the public via the institution website up to the year 2019. Upon completion of our systematic search of the institutions (n = 266), the data for coding were comprised of 64 institutions. This indicates that 24% of the institutions had publicly available authorship policies.

### Coding Procedures

The coding guide was developed based on the extant literature reviewed above and relevant themes that emerged during initial coding of policies. Five coders were used to independently code the institutional data. Coders met weekly to resolve any questions that emerged during the coding process. Coders used the following inclusion criteria when identifying institutional guidance/policies to be included for data analysis. First, institution-level ownership was defined as having a publicly accessible policy at the school/college-level and higher within the institutional hierarchy. Coders used two qualifications for this criterion, with only one qualification necessary for inclusion (i.e., both qualifications were not necessary for data inclusion). The webpage or location of the authorship guidance could signal institution-level ownership of the authorship guidance. For example, authorship guidance on an institution’s Office of Research or Graduate School webpage signals the institutional level, as these levels hierarchically transcend any college or school in an institution. Second, the institutional level could be represented by clear institutional ownership displayed and/or stated in the authorship guidance (e.g., the institution’s endorsing/approving body, institution-specific procedures for dispute resolution, etc.).

Note that neither an institution’s Responsible Conduct of Research (RCR) training webpage, nor the institution's library webpage, sufficiently signaled institution-level ownership on their own; thus, policies/documents from RCR and library pages were only included if they also met the second qualification listed above. Any data that required both qualifications were discussed among coders to reach consensus for inclusion. Upon concluding that an authorship policy was at the institutional level, coders identified whether the policy specified authorship criteria, recommended when to discuss authorship, mentioned authorship dispute resolution, and discussed faculty-student collaboration. As a reliability check, two authors re-coded 10% of articles and found no discrepancies.

To investigate how the policies outline dispute resolution, we examined the text of each policy related to authorship disputes. One author extracted text describing dispute resolution and a second author reviewed the text to ensure relevancy. Through this process, we coded the length of the policy, Carnegie classification, presence of medical school, as well as several elements of the policy itself. Several coding categories were developed a priori based on the relevant literature, but the team also added categories through an inductive process while coding. Specifically, we wanted to identify whether the policy mentioned student-faculty collaboration or collaboration across multiple institutions and recommendations for resolving authorship disputes. We coded whether the policy recommended resolving the dispute within the author team and/or consulting others, such as the department chair, ombuds, Dean, Provost, or Research Integrity Office. One author coded all policies and a second author re-coded 20% of policies (*N* = 8) as a reliability check. Data tables for this work can be found on the Open Science Framework at https://osf.io/5cz6m/?view_only=7dc1fdd53de54a58a615c11366202a4e.

### Data Analysis

In order to address our research questions, we first calculated frequencies and bivariate correlations to examine whether Carnegie classification (i.e., R1 or R2 designation) or medical school status (i.e., a university had a medical school versus not) related to the likelihood of a public, institutional authorship policy. Of the institutions that had public authorship policies, we examined the depth of the policy by Carnegie classification and medical school status. Here, we determined the depth of the policy by summing across the four coding categories mentioned above—specification of authorship criteria, recommendations for when to discuss authorship, mention of dispute resolution, and discussion of student-faculty collaboration. Each of these categories was assigned a value of 1.00, resulting in a maximum value of 4.00 for the depth of the policy. To analyze the extent to which universities discussed authorship dispute resolution, we first identified the word count of the text describing dispute resolution. Then, we calculated the mean and standard deviation of the word counts and compared across institutions. In terms of the content of the policies, we calculated frequencies for each coding category described above (e.g., recommendation to resolve the dispute within the author team and/or consult others).

## Results

### Authorship Policies

Of the 266 universities identified in the Carnegie classifications, 24% (64 Universities) had an authorship policy that was available to the public on their website and met our inclusion criteria. Within this pool of universities, about 80% were classified as R1 universities versus R2 universities (R1 = 51 universities, R2 = 13 universities) and about 83% had a medical school (medical school = 53 universities, no medical school = 11 universities). We also calculated the bivariate correlations between Carnegie classification (R1 = 1 versus R2 = 0), medical school status (university has a medical school = 1 versus not = 0), and authorship policy (policy = 1 versus no policy = 0). We found that medical school status demonstrated the strongest effect on the likelihood for a university to have a public-facing authorship policy (*r* = 0.45, *p* < 0.001), with Carnegie classification demonstrating a slightly weaker effect (*r* = 0.34, *p* < 0.001).

We also examined the characteristics of each authorship policy by focusing on the following four aspects: (1) specifying authorship criteria, (2) recommending when to discuss authorship, (3) mentioning dispute resolution, and (4) mentioning faculty-student collaboration. Within the pool of universities with a public-facing authorship policy, the most common policy characteristic was specifying authorship criteria, with 94% of universities adopting this policy characteristic. Next, 66% of universities recommended when to discuss authorship and 64% of universities mentioned dispute resolution. The least common policy characteristic was mentioning faculty-student collaboration, with only 54% of universities adopting this policy characteristic (see Table 1). We calculated the total depth of each university’s authorship policy across all four characteristics (see the Methods section for a description of this analysis) and found that, on average, public-facing authorship policies demonstrate a depth of *M* = 2.78, *SD* = 1.24 (on a one to four scale) (see Table 2).

Taking a more nuanced perspective, we also examined the above policy characteristics using both the R1/R2 and medical school status distinctions. In terms of Carnegie classification, R2 Universities demonstrated a slightly stronger average for the depth of the authorship policy (R1: *M* = 2.75, *SD* = 1.26, R2: *M* = 2.92, *SD* = 1.19), although the mean difference was not significant. Additionally, R2 universities were more likely to recommend when to discuss authorship (R1 = 63% versus R2 = 77%) and mention faculty-student collaboration (R1 = 53% versus R2 = 62%). In terms of medical school status, universities without a medical school demonstrated a stronger average for the depth of the authorship policy (Medical school: *M* = 2.66, *SD* = 1.26, No medical school: *M* = 3.36, *SD* = 1.03), and this mean difference was marginally significant (*t* = 1.74, *p* < 0.10). As such, universities with no medical school demonstrated the strongest depth of authorship policy. Additionally, universities with no medical school were more likely to include all four policy characteristics when compared to universities with medical schools. For a complete depiction of these findings, see Table 3 (note that we do not include differences between R1 and R2 Universities as the mean difference was not significant).

**Table 1:** Frequency of authorship policy by university

Authorship Policy	Carnegie Classification		Medical School Status	
	*R1*	*R2*	Medical School	No Medical School
	*n* = 131	*n* = 135	*n* = 114	*n* = 152
Has a policy	51 (38.90%)	13 (9.60%)	53 (46.50%)	11 (7.20%)
Policy Mentions Dispute Resolution	35 (26.72%)	7 (5.19%)	35 (30.70%)	7 (4.61%)
No policy	80 (61.10%)	122 (90.40%)	61 (53.50%)	141(92.80%)

*n* = 266 total Universities

**Table 2:** Bivariate correlations

Variable	1	2	3
Carnegie classification	–		
Medical school status	0.44^*^	–	
Authorship policy	0.34^*^	0.45^*^	–

*n* = 266 Universities. ^*^p < .001. Carnegie classification: 0 = R2 University, 1 = R1 University. Medical school status: 0 = no medical school, 1 = medical school. Authorship policy: 0 = no policy, 1 = has a policy

**Table 3:** Authorship policy characteristics

Policy Characteristics	All Coded Universities	
	(*n* = 64)	
	Frequencies	
Specifies authorship criteria	94%	
Recommends when to discuss authorship	66%	
Mentions dispute resolution	64%	
Mentions faculty-student collaboration	55%	
	Mean (Standard Deviation)	
Total depth of policy	2.78 (1.24)	
Policy Characteristics	Medical School	No Medical School
	(*n* = 53)	(*n* = 11)
	Frequencies	
Specifies authorship criteria	93%	100%
Recommends when to discuss authorship	62%	82%
Mentions dispute resolution	62%	73%
Mentions faculty-student collaboration	49%	82%
	Mean (Standard Deviation)	
Total depth of policy	2.66 (1.26)	3.36 (1.03)

*n* = 64; The total depth of policy ranges from 0 (no identified policy characteristics) to 4 (all identified policy characteristics)

### Dispute Resolution Policies

Of the 64 authorship policies identified in our sample, 42 mentioned dispute resolution (61%). The majority (*n* = 35, or 83%) of these policies came from universities with a medical school or from the medical school directly, which aligns with the proportion of medical schools in the broader sample of universities featuring authorship policies. Of these dispute resolution policies, only 7 (about 17%) came from R2 universities. On average, the section regarding dispute resolution consisted of 273 words (*SD* = 419.59), ranging from 23 to 2048 words.

In terms of the content of the policies, the majority of these policies (*n* = 36, or 86%) recommended that authors first attempt to resolve disputes among themselves. For continuing disputes, many policies recommend consulting a department chair (*n* = 32, or 76%) or Dean (*n* = 31, or 74%). Less commonly mentioned resources for dispute resolution included the Provost (*n* = 9, or 21%), the Ombuds (*n* = 7, or 17%), and the Research Integrity Officer (*n* = 9, or 21%). Very few policies (*n* = 5, or 12%) mentioned authorship disputes where multiple universities were involved. Similarly, only 10 policies (*n* = 24%) mentioned resolving authorship disputes in teams consisting of both faculty and students. Additional details regarding the content of dispute resolution recommendations can be found in Table 4.

**Table 4:** Dispute resolution policy characteristics

Characteristic	Frequency	Universities with dispute resolution policies (*n* = 42) %	R1 and R2 universities with authorship policies (*n* = 64) %
Resolve Internally First	36	86	56
Department Chair	32	76	50
Dean	31	74	48
Provost	9	21	14
OMBUDS	7	17	11
RIO	9	21	14
Committee	11	26	17
Student/Faculty Disputes	10	24	16
Multiple Institutions	5	12	8
Medical School	35	83	55
R1	35	83	55

*n* = 266 total Universities

## Discussion

The current review identified that only 24% of the 266 R1 and R2 institutions had public, institution-level authorship policies. Given the established importance of authorship (Rennie and Flanigan 1994; Wilcox 1998; Macrina 2011), as well as the potential for ambiguity and conflict over authorship (Smith et al. 2019a; Marušić et al. 2011; Birnholtz 2006; Gómez-Ferri et al. 2019), this appears to be a critical oversight on the part of the most research-productive institutions in the United States. Our work addresses the gap in understanding problematic authorship practices by analyzing the publicly available authorship policies from 64 R1 and R2 institutions, with a particular focus on author dispute resolution. We now discuss the implications of our findings for institutional authorship policies in general and, more specifically, guidance for authorship disputes.

### Authorship Policies

Of the 266 universities identified in the Carnegie R1 and R2 classifications, only 24% (64 universities) had an institution-level authorship policy. This number seems surprisingly low given the primary focus on authorship and publishing as key to academic career success. Established policies can be advantageous for institutions as they guide employee expectations and decision making, keep higher-power individuals accountable, and inform employees on where to turn for help when faced with a problem. As publications are considered the coin of the realm for academics, the advantages that accompany the use of an established policy seem particularly important for authorship practices due to the potential for conflict and abuse surrounding such a high-stakes aspect of the academic job.

The majority of universities with a public-facing authorship policy were classified as R1 institutions (80% at 51 universities) and/or had a medical school (83% at 53 universities); however, grounded in our correlational analyses, medical school status appears to be more strongly related to the likelihood for having an institutional authorship policy. This is consistent with the tradition among medical schools to acknowledge authorship practices via such organizations as ICMJE. While the prevalence of institutional authorship policies may be greater for universities with a medical school and/or high research activity, this does not necessarily speak to the quality of the policy. In fact, institutional authorship policies in our sample demonstrated, on average, a depth of 2.78 out of 4.00, suggesting there is room for improvement. Across all included institutions, the most common policy component was specifying authorship criteria (at 94% of universities), which aligns with published guidelines from ICMJE and other related organizations as discussed above.

The least common policy component was related to collaboration between students and faculty, with only 55% of institutional authorship policies mentioning this issue. Students represent a vulnerable population in terms of authorship ethics, as they do not have the same rights as faculty or the same security in their position at the university. Due to the dependence of most graduate students on their faculty mentor, students may not be comfortable addressing authorship issues when they arise. Further, faculty mentors often control publication and research funding opportunities for students, both of which are vital to student success in school and post-graduation. Institutional guidance for authorship in faculty-student collaborations has the potential to reduce the likelihood for students to be taken advantage of and address any problematic “hidden curriculum” regarding authorship practices. Further, making issues regarding authorship ethics visible to students helps them build awareness of the matter and practice habits of transparency and fairness in the distribution of authorship.

Interestingly, universities without a medical school demonstrated the highest quality institutional authorship policies as they were more likely to include all coded policy components. Indeed, 82% of universities without a medical school included a policy component on authorship practices for student-faculty collaboration (compared to 55% mentioned above for all universities and 49% for just universities with medical schools). This may be due to universities with medical schools more heavily prioritizing the standards put forth by organizations such as ICMJE. For example, while ICMJE provides materials around what constitutes an author and issues such as ghost, gift, and guest authorship, they do not specifically discuss other types of authorship issues, such as dispute resolution or student-faculty collaboration. Thus, universities that emphasize standards from the ICMJE may be less likely to provide authorship guidance stemming from issues that are not discussed by the ICMJE.

### Authorship Disputes

#### Dispute Prevention

Ideally, authorship disputes could be avoided entirely through the use of prevention techniques, thereby ensuring that communication of research findings is not delayed, appropriate credit is assigned, and productive teamwork is not harmed. One effective tool for preventing disputes is clear and early communication between team members regarding authorship expectations, and most policies recommended this practice (63% of included policies). For example, one policy notes that “as early as possible in the research process, Principal Investigators should inform collaborators of the requirements for authorship of any manuscript that will report results of the project.” This step can be easily overlooked; however, an agreement amongst all authors can remind authors of their obligations and prompt revisitation of authorship lists as individual roles evolve throughout the research process. A few policies recommended written agreements, which can be especially beneficial in providing evidence of past agreements for authors in lower-power positions. However, few dispute resolution sections of policies mentioned prior agreements (verbal or written) as a recourse for resolving dispute, though this practice is frequently recommended (Frassl et al. 2018, 3; Vicens and Bourne 2007; COPE 2016). We found no other formal or informal attempts to prevent authorship disputes in the policies surveyed.

#### Dispute Resolution

Only 16% of R1 and R2 universities (*n* = 42) have dispute resolution policies. We noted both substantial overlap and distinctions between policies from different institutions. For overlap, text comparison revealed that some policies were almost identical as noted through acknowledgements of other source policies. Other policies were fairly idiosyncratic, which suggests that these policies arose in response to particular events at each institution. The small number of dispute resolution policies is surprising given (a) the prevalence of disputes previously discussed and (b) the fact that journals often avoid resolving disputes. In cases where prevention fails, it helps to have an institutional dispute resolution process for several reasons. First, public policies offer transparency regarding processes, which benefits all parties by establishing shared expectations. Second, the process of building a policy, provided it incorporates a diverse and inclusive set of perspectives, is better able to accommodate the contexts in which such disputes arise. Third, a formalized process can often allow for reasonable resolution without disputants resorting to damaging public or social media complaints, or silencing the complaints of those in lower-power positions. These factors strongly point to the importance of institutional policies to resolve authorship disputes.

Aside from the overlap in policies using almost identical verbiage, a range in dispute resolution provisions emerged when examining the 42 dispute resolution policies identified in our study. The majority of policies with dispute resolution provisions (*n* = 36 out of 42, 86%) suggested that authors first try to resolve disputes among themselves, or involve the department chair (*n* = 32, 76%) or Dean of the college (*n* = 31, 74%). These are reasonable first steps with smaller authorship teams within departments or colleges, but when institutional teams reach across colleges, this guidance may not be helpful without specifying how Deans can work together on authorship disputes. Other potential approaches were anticipated in some policies; for example, some recommended involving a Provost (*n* = 9, 21%), Ombuds (*n* = 7, 17%) or RIO (*n* = 9, 21%). Some policies (*n* = 11, 26%) reference the formation of a dispute resolution board or committee, but for the most part, these policies do not offer many details concerning the composition of the committee or its specific role.

In addition, there seems to be no best-practice consensus on *inter-institutional* authorship disputes. Authorship is increasingly multi-disciplinary, multi-institutional, and trans-national (Adams and Gurnsey 2018; Hsiehchen et al. 2015). Very few policies explicitly addressed this potential issue (*n* = 5), leaving multi-institutional author teams without guidance on how to resolve disputes. One policy suggested that a “Publication Subcommittee representing all Investigators should be established at the beginning of any multi-center study for the purposes of expediting, coordinating, and monitoring the paper-writing processes.” While this recommendation may prove useful for larger project teams that span across multiple universities, there are many circumstances where small authorship teams span multiple universities and disciplines. There were no recommendations for resolving authorship disputes in the latter circumstance within our sample.

A few policies specifically indicated that if a dispute is sent to a dispute committee with the permission of all parties, then any party has the right to make the committee’s (non-binding) recommendations public. In such cases, some parties may, rightly or wrongly, attempt to adjudicate the dispute in the court of public opinion. Policies often indicated that authorship disputes do not qualify as research misconduct, but many institutions also include the Research Integrity Officer or the department of research integrity in the hierarchy of potential recourse, which may send mixed messages to those using the policies. If journals routinely remand authorship disputes to institutions for resolution, universities with no policies or vague policies may lack the resources to resolve these disputes, delaying publications and thus hampering the dissemination of research findings and publication credit for their faculty and students. Seen with this lens, it is ironic that one university’s policy notes the potential for involving a journal editor in cases of disputed authorship.

Notably, policies seemed to lack discussion of enforcement. While some indicated, for example, that a dean’s decision about authorship would be “final” or “decisive,” there was no indication of consequences should authors not abide by that decision. Several institutions mention possible “sanctions,” but rarely specify the exact sanctions and who might impose them. In sum, explicit discussion of enforcement of authorship policy was rare. Without enforcement or consequences, it is difficult to determine what impetus institutions will have to publicize their policies or what incentives faculty and staff may have to learn about and follow a policy. Moreover, individuals in lower-power positions have the most to lose by reporting authorship abuse, yet only one policy surveyed specifically prohibited retaliation for authorship disagreements and complaints. Presumably, most institutions already have a non-retaliation policy. If so, authorship dispute resolution policies should explicitly invoke it in order to ensure that potential disputants feel protected from reprisal. Overarchingly, the majority of dispute resolution policies lacked sufficient specificity for the policy to be of much use. The wide diversity of recommendations and lack of evidence for consensus about best approaches to resolving authorship disputes indicate that there is as yet no consensus on best practices for intra-institutional disputes. Thus, more work on productive approaches to authorship dispute resolution policies is needed.

### Study Limitations

While we believe this work takes an important step forward in examining the prevalence and content of institutional authorship policies, there are several limitations to our study. One limitation is our reliance on university websites to obtain authorship policies. Here, access to authorship policies may be subject to technical issues. For example, any potential search hits for an authorship policy had to be accompanied by a functional web link to the actual policy. Any potential authorship policies with broken links (i.e., it was not possible to access the policy via the university’s website) were not included in our study. Additionally, some universities may have institution-level authorship policies, but not provide them publicly on their websites. Any such policies were not included in our study. Thus, interpretation of our findings should be from the perspective of universities providing *public-facing* authorship policies. However, we suggest that having public-facing authorship policies (rather than policies merely accessible within the institution) are important for stakeholders. For example, inter-institutional collaborations are already common, so collaborators from other institutions could access policies for all members of a research team if needed.

### Recommendations for Future Policies

Our survey found many policy components to be both common and supported by the literature in this area. However, it did not reveal broad consensus on the fine-grained details of institutional authorship policies (such as dispute resolution practices). Below, we share our holistic interpretation of good practices developed from the survey and literature review that will guide our own institution’s authorship policy process.

#### Reasons for Having a Policy

An institutional authorship policy is important for creating and supporting an ethical culture. Given the increasing potential for authorship disputes, and the lack of alternative processes for addressing them, a clear policy will provide predictable mechanisms that may help to ease tensions between collaborators. Moreover, normalizing authorship discussions early and often in collaborative processes may have additional benefits for students and junior faculty, who may lack the ability to raise these issues themselves. By making authorship criteria transparent, graduate students may learn the lessons we want them to learn about good authorship decisions, rather than learning—and then later reproducing—a “hidden curriculum” whereby the power to make authorship decisions is abused. Thus, it is clear to us that a well-publicized institutional authorship policy would be a benefit for all institutions.

#### Elements of a Policy: Justification, Applicability, Criteria, Prohibitions

Our survey found several common elements in extant policies that we intend to include in our own policy development:

- Justification: Most policies include an introductory section communicating the reasons for an authorship policy, including protecting the integrity of research and careers, and supporting and promoting healthy communication. Empowering those potentially affected by an authorship policy with knowledge about why the policy is important (and communicating those reasons in publicity efforts) is another good way to promote ethical culture.
- Applicability: A policy should also communicate the scope or applicability of the policy. For example, will faculty, students, and certain staff be subject to the policy, and under what conditions? Because authorship disputes between faculty and graduate students can occur, policies such as faculty or graduate student grievance processes would likely not be able to address a faculty/student dispute.
- Criteria: Although policies frequently presented general or commonly accepted criteria for authorship, they were almost universally extremely careful to acknowledge and defer to wide disciplinary diversity in authorship norms (including author ordering). Many mentioned (without endorsing) international standards such as those from the International Council of Medical Journal Editors (ICMJE) or the Committee on Publication Ethics (COPE). By communicating both general criteria for authorship (e.g., making a significant intellectual contribution) as well as referring to a variety of existing norms, this element of a policy can uphold ethical culture and inform institutional members of resources to which they can turn for more information. For this reason, it also seems wise to include, as an appendix to a policy or in some other form, a set of references about authorship issues.

Policies also frequently encouraged collaborators to articulate together which standards they plan to follow through open, two-way conversation resulting in oral or written agreements, with the latter being preferred. Other forms of documentation, such as creating a project on the Open Science Framework and listing author order at the beginning of a project, can serve a similar purpose (https://osf.io/). Such steps facilitate an initial conversation even if authorship order ultimately needs to be changed based on contributions. Several policies in our survey suggested referring back to such agreements to resolve disputes, and published literature similarly suggests that early discussion and formalized authorship agreements can be helpful (Marušić et al., 2014). Policies should also note that authorship can change in the course of research and invite collaborators to revisit authorship determinations as warranted throughout the lifecycle of research production and dissemination. These practices benefit students in particular by educating them in good publishing practices and offering a more transparent approach to assigning authorship credit. Finally, the practice in several policies of explicitly assigning to the communicating author the responsibility to discuss authorship collegially seems to be a beneficial policy element.

- Prohibitions: One way in which several policies were quite specific was in explicitly denouncing practices like ghost, guest, or gift authorship. Because there is more agreement about prohibited practices, and because explicit denunciation of these practices in a policy potentially arms a wronged party, this seems to be a beneficial policy element as well.

#### Dispute Resolution

Authorship policies implicitly acknowledge that disputes are predictable and likely. We think that no authorship policy will be complete without addressing possible disputes, especially given the lack of alternative resolution mechanisms we outlined above. Given the high stakes attached to authorship decisions, a transparent, predictable process based in policy may help both to avoid disputes in the first place and address them if they arise.

Policies we surveyed overwhelmingly recommended trying to resolve disputes initially at the lowest hierarchical level possible, e.g., between the collaborators, with the help of the chair of a department if the dispute concerns only members of that department, or with the dean of a college if the dispute stretches across units. Because this is likely to resolve at least some disputes, we think these are good initial steps. Common next steps among policies were to consult a “neutral third party,” including an Ombuds, or occasionally a Grievance Committee, Dean, Vice-Provost, Provost, Research Integrity Officer, General Counsel, or Vice-President of Research. Some policies also included bodies unique to that institution. A few policies indicated that if a student is involved, the Chair of the Dissertation Committee, the Director of Graduate Studies, or the Graduate Student Grievance Committee might be included in an informal resolution process. While it may be helpful to include an interim step after early attempts at resolution and before invoking a more formal process, we found no consensus regarding whom to invoke as a neutral third party.

In our survey, only eight institutions with dispute policies mentioned a final, formal step of convening a committee or panel to consider disputes, and only five of them (Clemson, Duke, Emory, George Mason, and Louisiana State) outlined specific processes for this. The others indicated that a committee was one option among others, and in one case only provided for a student committee. Although a very small proportion of the 266 surveyed institutions include this step, we think that policies should acknowledge the possibility of protracted and challenging disputes that cannot be resolved by the steps outlined above, and provide a more formal resolution process that encompasses disputes between any combination of student, faculty, and staff.

Faulkes (2018) has recommended alternative dispute resolution mechanisms, either at the level of a journal editorial office or through independent third parties who may serve as mediators on a fee-for-service basis. However, we think there are benefits to a process that remains at the institution. Our tentative plan is to provide for an Authorship Dispute Committee, with membership drawn from various institutional areas such as the Ombuds for faculty and students, administrators, and content area experts as needed. A few policies include very detailed steps regarding written notice, time frame, and appeals. This has the benefit of providing a very clear process, but also makes for a much more cumbersome policy that is consequently less likely to be read, so we have not yet reached a decision on this step.

#### Enforcement

The most challenging aspect of an institutional authorship policy may be enforcement. Most policies did not make any reference to enforcement, and those that did were not explicit about how this would be achieved, instead simply stipulating that a certain decision would be “final.” This is an area needing significant additional analysis (including legal analysis) because it involves balancing faculty rights to academic freedom and due process against an institution’s obligation to resolve authorship disputes.

As a result, our current plan is not to include any provision for enforcement.

#### Publicity

Once established, an institutional authorship policy should be publicized across campus, and training on good authorship practices should be offered to both faculty and graduate students. Faculty would benefit from help in initiating difficult conversations with graduate students, and graduate students could use the policy to raise what might otherwise be a difficult topic with faculty supervisors. It is important to acknowledge that publicizing such policies can predictably flush already-existing disputes and tensions into the open, so it will be critical to have mechanisms in place for that eventuality prior to publicity efforts.

We hope that as more institutions develop authorship policies, additional research will bring to light evidence for best practices. In the meantime, we offer these ideas to others as our considered opinion developed by months of immersion in existing policies.

## Conclusion

This paper addresses a critical lack of knowledge regarding the role of the university in guiding current authorship practices by examining the prevalence and content of authorship policies from R1 and R2 Universities. We found that only 24% of these universities have public-facing, institution-level authorship policies. In addition, we found that authorship policies in general varied widely in terms of their content, with specific dispute resolution policies also varying in length and specificity. Based on our examination of these policies and the extant literature, we present recommendations to encourage the adoption of thorough, effective, and enforceable institutional guidelines regarding authorship.
